# Exploration of Hand Grasp Patterns Elicitable Through Non-Invasive Proximal Nerve Stimulation

**DOI:** 10.1038/s41598-017-16824-1

**Published:** 2017-11-29

**Authors:** Henry Shin, Zach Watkins, Xiaogang Hu

**Affiliations:** Joint Department of Biomedical Engineering, University of North Carolina at Chapel Hill, NC and North Carolina State University, Raleigh, NC USA

## Abstract

Various neurological conditions, such as stroke or spinal cord injury, result in an impaired control of the hand. One method of restoring this impairment is through functional electrical stimulation (FES). However, traditional FES techniques often lead to quick fatigue and unnatural ballistic movements. In this study, we sought to explore the capabilities of a non-invasive proximal nerve stimulation technique in eliciting various hand grasp patterns. The ulnar and median nerves proximal to the elbow joint were activated transcutanously using a programmable stimulator, and the resultant finger flexion joint angles were recorded using a motion capture system. The individual finger motions averaged across the three joints were analyzed using a cluster analysis, in order to classify the different hand grasp patterns. With low current intensity (<5 mA and 100 µs pulse width) stimulation, our results show that all of our subjects demonstrated a variety of consistent hand grasp patterns including single finger movement and coordinated multi-finger movements. This study provides initial evidence on the feasibility of a proximal nerve stimulation technique in controlling a variety of finger movements and grasp patterns. Our approach could also be developed into a rehabilitative/assistive tool that can result in flexible movements of the fingers.

## Introduction

After an injury to the central nervous system, such as a stroke or a spinal cord injury, a majority of individuals have impairments in their ability to voluntarily activate their muscles, manifested as a weakness in both their upper and lower extremities^1–4^. Among the different motor and sensory functions involved in daily activities, regaining hand grasp function is considered a top priority in improving the quality of life for individuals with paralysis^5^.

In order to help restore some of these lost hand functions, a wide variety of functional electrical stimulation (FES) techniques have been developed^6–8^. However, the utility of FES has been limited due to several key factors. First, with electrical stimulation, motor units are believed to be recruited in a reverse physiological order, in that the large and fast-fatigable motor units are recruited earlier. Although other factors, such as the electrode location and the relative location of the motor points in the imposed electrical potential field, can also influence the recruitment order^9^, which can lead to random recruitment. Nevertheless, the control of graded muscle forces through stimulation tends to be difficult, and rapid fatigue onset is also common. Secondly, most of the stimulation approaches use a large diameter electrode pad placed on the skin surface in proximity to the innervation zones of the targeted muscles. These techniques can typically only access a limited number of muscles, most of which are superficial muscles^10^. For example, most stimulation methods only target extrinsic finger muscles during the stimulation, which can lead to unnatural movement kinematics^11,12^. The large size of the electrode pad also limits the selectivity of muscle activation, and therefore, the elicited movements are largely gross hand opening and closing, rather than dexterous finger movements. Lastly, in order to elicit functionally meaningful muscle forces, the delivered current intensity tends to be uncomfortably high.

Various recent developments in FES techniques have sought to address these issues. For example, a spatially distributed multi-pad electrode grid has been used to distribute the stimulus current to different regions of the muscle belly^8,13,14^. This approach has been shown to be able to delay muscle fatigue onset, reduce discomfort, and increase the selectivity of muscle activation. However, since the stimulation targets the motor points, the required current amplitude is still high (typically well above 10 mA). Alternatively, invasive procedures involving implantable electrodes with a direct interface to the peripheral nerves have also been developed^6,15,16^. Specifically, electrode array cuffs or penetrating electrode grids can be implanted to the proximal segments of the peripheral nerve innervating the targeted muscles. Low amplitude current delivered to selective nerve fibers can activate specifically targeted muscles to elicit precise finger movements. However, this approach requires invasive surgical procedures, and the long-term stability of the implants still needs improvement.

Accordingly, the purpose of our current study was to explore the feasibility of using a transcutaneous nerve stimulation technique to elicit dexterous finger grasp movements. Specifically, we delivered low level current to the nerves proximal to the elbow joint, and quantified the elicited range of motion of the individual joints in the hand. Peripheral nerve stimulation for controlling finger flexion targets two nerves: the ulnar and median nerve bundles. These nerves innervate and bilaterally divide both the intrinsic and extrinsic flexor muscles of the hand based on the different fingers. The median nerve innervates the index and middle fingers and partly the ring finger, whereas the ulnar nerve innervates the little finger and partly the ring finger. These nerves are relatively superficial near the biceps brachii muscle, and therefore electrical stimulation in this region can be used to activate the distal wrist and finger flexors. Depending on the location of these two nerves in the imposed electrical potential field, different portions of the median and/or ulnar nerves can be activated, which can lead to isolated or coordinated finger movement. Our results show that this stimulation technique is able to generate movements in the fingers both individually and in coordinated groups. The findings can facilitate the development of non-invasive nerve stimulation techniques that can help individuals with hand weakness/paralysis in regaining functional finger movement.

## Methods

### Subjects

**Figure 1:**
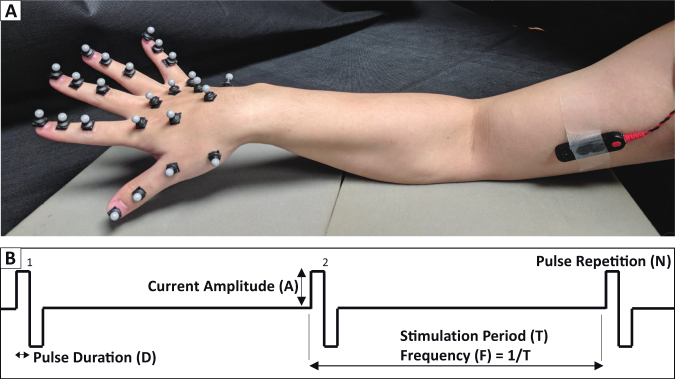
Overview of Experimental Setup. (**A**) Optical Tracking markers were placed on the hand and the bar electrode was placed over the region of skin on the inner face of the biceps. Positioning of the hand is solely for illustration purposes, i.e. displaying the marker setup and the stimulation electrode placements, and is not the actual experimental positioning. (**B**) Sample stimulation train. Illustration of the different stimulation parameters. Current amplitude and pulse frequency were the main two parameters that were adjusted between trials.

Eight healthy subjects (6 males and 2 females with 21–34 years of age) without any known neurological disorders participated in the study. For each subject, electrical stimulation was delivered transcutaneously, and the resultant finger motions were recorded using a motion tracking system (Fig. 1A). All subjects gave informed consent with protocols approved by the Institutional Review Board of the University of North Carolina at Chapel Hill. All the methods were performed in accordance with the relevant guidelines and regulations of the Institutional Review Board of the University of North Carolina at Chapel Hill.

### Experimental Setup

#### Stimulation Generation

All electrical stimulation was delivered by a multi-channel stimulator (STG4008, Multichannel Systems, Reutlingen, Germany). The stimulator was controlled using a custom-made MATLAB user interface which generates stimulation trains. A sample stimulation train is shown in Fig. 1B, outlining the basic parameters which can be specified in the MATLAB interface. In this study, the pulse duration was kept constant at a 100 µs duration, but the Current Amplitude, Frequency, and number of Pulse Repetitions were varied in the survey of stimulation-induced motions.

#### Finger Motion Tracking

The resultant motion of the fingers elicited by the electrical stimulation was tracked using an 8-camera Optitrack System (Natural Point, Inc, Corvallis, OR). Each subject’s right hand was first prepared for motion tracking by attaching 6 mm IR reflective markers to each joint of the fingers and the thumb, as well as over the distal phalanx and the metacarpals of each digit (Fig. 1A). In total, 24 markers were placed on the hand and recorded using the companion motion capture software (Motive, Natural Point, Inc, Corvallis, OR). The 3D position data of each marker were resolved in real time by the Motive software and were sampled at 100 Hz. The motion tracking recording was synchronized to the start of the output of the stimulator through a synchronization pulse.

### Procedures

The subjects were seated prior to the initial setup. The hand was placed near the center of the motion area and the wrist was rested over foam blocks which kept the hand comfortably elevated while allowing all the markers to be seen by the cameras. The forearm was maintained in a neutral position with the hand opened and relaxed. The subject was asked to reopen the hand after stimulations, but otherwise remained completely relaxed and still. A bar stimulation electrode (4 cm inter-electrode distance) with conductive paste was placed on the skin, over the ulnar/median nerve bundles located laterally beneath the short head of the biceps brachii. This location was readily found by asking subjects to activate their biceps, after which the electrode was placed spatially underneath the bulge of the biceps muscle. Although this general anatomical location was consistent across subjects, the exact position was not quantifiable due to inter-subject variations in anatomy and the lack of precision in measuring the exact orientation of the electrode in space. Using a stimulation train at 4 mA and 25 Hz (and constant 100 µs duration), the experimenter manually held the electrode and modified the positioning of the electrode tosearch for locations in this area which induced noticeable movements of the hand. The searching procedure typically involved small changes in the medio-lateral orientation of the electrode while still attached to the skin, or lifting and shifting of the electrode either distally or proximally (less than ~1 cm) and repeating the previous process until activation of the hand was seen. Once a stimulation location with a consistent, repeatable hand motion was obtained, the electrode was securely attached with medical tape and the hand-held electrode orientation was mimicked as best as possible with constant pressure applied with a custom-made clamp. An initial stimulation train at 4 mA, 25 Hz, and 1 second duration was delivered and repeated 10 times. Each stimulation train was spaced by 2–5 seconds of rest for the subject to return to the initial neutral position. This set of 10 stimulation trains with rest will hereby be referred to as a single trial. The stimulation parameters of each trial were saved and delivered while simultaneously recording all the resultant positions of each finger joint. Given that the main focus of this study was to quantify the hand grasp patterns, the wrist and forearm positions were not recorded. Several trials with variations in the stimulation parameters (i.e., current amplitude and pulse frequency) were tested with each stimulation location. Current amplitude across all the subjects ranged from 2 to 8 mA (mean ± standard deviation: 4.23 ± 1.22 mA). The pulse frequency was mainly tested at 25 and 50 Hz in a majority of the subjects. Some subjects were also tested at other frequencies from 10 to 100 Hz, time permitting. The main purpose of these parameter changes was to modulate the strength of the contraction. Since the focus of the study was to survey which finger motions could be elicited, the parameter changes were geared towards observing if different parameters changed the activated motion, rather than a systematic investigation of the parameter space. The stimulation electrode location was then shifted to identify a different stimulation location that could elicit a unique repeatable hand motion. Typically, 2–5 unique hand motions were found for each subject for a total of approximately 22 ± 8 stimulation trials per subject.

### Data Processing

**Figure 2:**
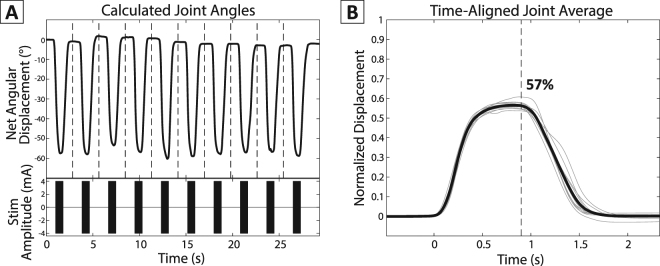
Time-aligned angular displacement. This sample data corresponds to the MCP joint of the Index finger of the same trial used in Fig. 3. (**A**) Single joint trace and corresponding stimulation train. The dashed line between the joint movements represents the splitting point of each stimulation. The stimulation train appears to be a solid block, but is actually a condensed version of Fig. 1A. (**B**) Time-aligned and normalized single joint motion with overlaid average. Thin lines represent the angle from individual stimulation train, and the thick line represents the averaged angle. The dashed line presents the peak angular displacement at the end of the stimulation train.

The motion tracking data were first processed with the Motive software and each marker’s position was manually matched and labelled to its corresponding joint. Portions of missing position data shorter than 0.5 seconds were interpolated using cubic splines to fill in any minor gaps. Recordings with more than 80% of the marker data missing for any joint was removed. This motion capture data was then exported to MATLAB, and the position of each marker over time was used to obtain the angular displacement of each of the 14 joints (2 × thumb + 3 × 4 fingers). The joint angle at each time point was calculated by applying inverse kinematics to the positions of each of the marker triplets, which corresponded to a joint. The net angular displacement was then calculated by subtracting the joint angle at the start of each stimulation. For each trial, the 10 repeated motions were time-aligned and the average value was then obtained for each joint (Fig. 2). The peak angular displacement of each joint was obtained by finding the angle value at the end of the stimulation train. These peak angles were then normalized by the range of motion (ROM) of each corresponding joint. For example, the average peak angular displacement for the sample joint in Fig. 2B was 50.7°, and was then normalized by its corresponding ROM, 90°, which resulted in the labeled 57%. The ROM values were taken from anatomical tables in the literature^17^. Any trials that did not show at least 20% ROM movement in any of the joints were removed before further analysis. The average variability (standard deviation) of each averaged set of 10 motions across all remaining trials was 4.327 ± 0.117 (mean SD ± standard error) degrees. Supplementary Figure S1 displays the average standard deviation for each subject.

### Data analysis

As the changed parameters did not noticeably alter the activation pattern of the set of trials within a single electrode location, all of the remaining trials (n = 126) were pooled together from each of the subjects for further analysis. Principle Component Analysis (PCA) and k-means clustering was utilized to identify different movement patterns. Specifically, PCA was used to identify the minimum number of principle components that explained 90% of the variance in the data set. In order to simplify the clustering, the individual joints of each finger were averaged together to represent the overall movement of each finger. This reduced the number of variables of each trial from 14 to 5 (3 joints per each finger, and 2 for the thumb). The average motion data was then classified into separate clusters based on the number of principle components, and an additional second level of PCA and k-means clustering was completed because each cluster was still comprised of different finger movement patterns. Different clusters were then manually identified as a type of multi-finger or single finger movement pattern.

### Statistical analysis

To examine whether particular joints (proximal vs. distal) were activated preferentially, the data from the four-finger grasp motion cluster (Cluster 1) were also compared between the individual joints [Metacarpophalangeal (MCP), Proximal Interphalangeal (PIP), and Distal Interphalangeal (DIP) joints] and the four fingers. A two-way (joint × finger) repeated measures ANOVA was performed. When the equal variance assumption was violated, a Greenhouse-Geissner correction was performed in the test. Post-hoc pairwise comparisons of the joints were used with Bonferroni adjustment. P < 0.05 was considered statistically significant.

### Data availability

The datasets generated and analyzed during the current study are available from the corresponding author on reasonable request.

## Results

### Relative Joint Motion for each trial

**Figure 3:**
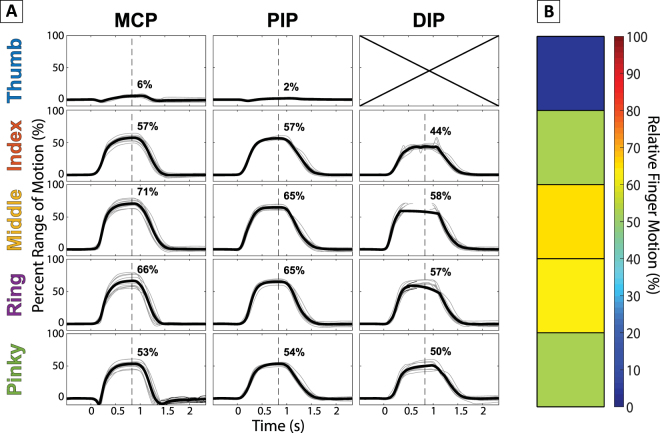
Sample Joint-Finger Motion Summary. The time-aligned, averaged, and normalized joint angles for all 14 joints were arranged corresponding to each finger and joint name. For further simplification, the relative motion of each finger was averaged again and displayed using a color gradient to simplify the total motion of the hand into a single column of information, with each cell representing a single finger.

**Figure 4:**
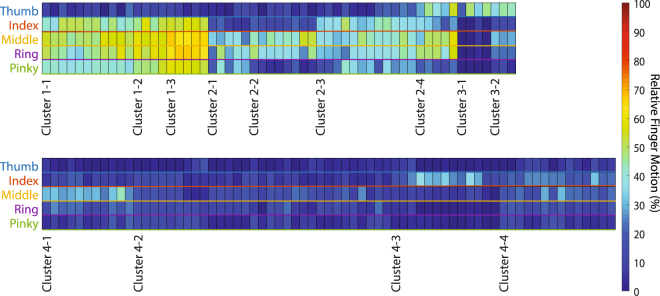
Clustered Relative Finger Motions. Summary figure for relative hand motion of all trials sorted based on two levels of k-means clustering. Cluster numbers (Main# - Sub#) indicate the first column (trial) that was categorized into the cluster.

Figure 3A shows a sample summary of all the 14 measured joints in a single trial taken from a representative subject. The peak average normalized angular displacement for each joint was then aggregated into a condensed representation of the relative joint motions for the entire hand in a given trial (Fig. 3B). Since all of the joint angles were normalized by its own maximum range of motion, these angles were visualized using a color gradient where blue was 0% range of motion and red was 100%. The compilation of this information results in the 2D “heat map” demonstrating the overall relative movement across all trials as shown in Fig. 4.

### Movement patterns

**Figure 5:**
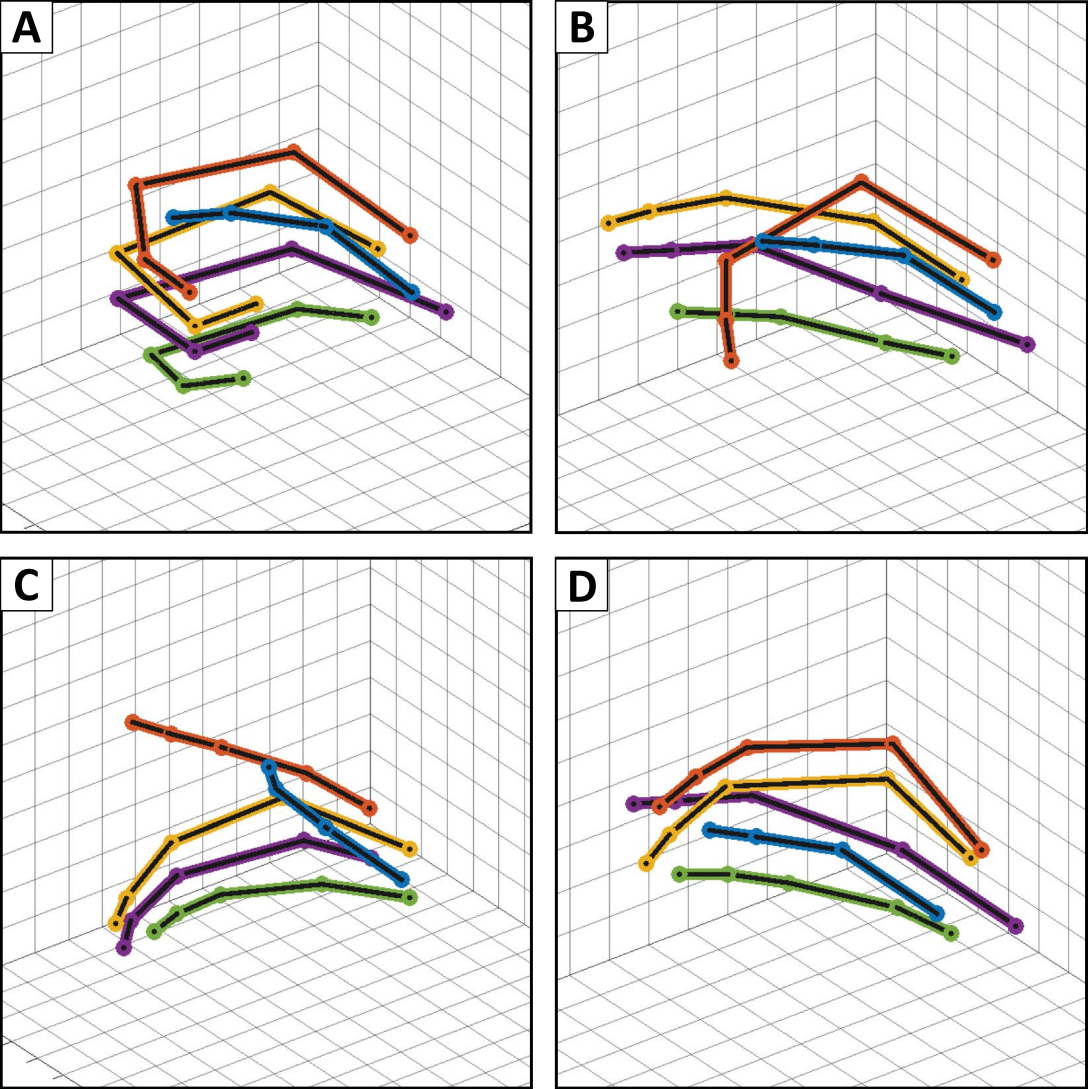
Example finger motions. (**A**) Four Finger Flexion (Cluster 1-3) (**B**) Index Finger Flexion (Cluster 4-3) (**C**) Middle-Ring-Pinky Flexion (Cluster 2-1) (**D**) Thumb-Index-Middle Flexion (Cluster 4-4).

Figure 4 shows the elicited finger motions grouped by the different clusters. Several 3D representations of the finger positions are also shown (Fig. 5) to further exemplify some of the distinguishable clusters. The most frequently observed pattern (20 out of 126) was a whole hand grasp pattern which involved the flexion of the four fingers (Main Cluster 1, Fig. 5A). The motion of the thumb was typically minimal during this whole hand grasp. The next biggest multi-finger clusters represents Index-Middle-Ring finger flexion with some sparse pinky movement (Clusters 2-3), isolated Middle-Ring finger flexion (Cluster 2-2), and Middle-Ring-Pinky finger flexion (Cluster 2-1, Fig. 5C). A multi-finger pattern which is less obvious from the clustering, but was still commonly observed, was Thumb-Index-Middle finger flexion, which we considered a pinching pattern (Cluster 4-4, Fig. 5D) Besides the coordinated multi-finger motions, the nerve stimulation was able to elicit independent single finger motions of the thumb (Cluster 3-1), index finger (Cluster 4-3, Fig. 5B), and middle finger (Cluster 4-1), however, independent ring finger and pinky movements were less common and were largely correlated with other finger movements. An expansion of Fig. 4 to show the motion of all the joints (Supplemental Figure S2) and exemplar videos corresponding to Fig. 5 are available in the Supplementary Materials.

### Joint Range of Motion Comparison

**Figure 6:**
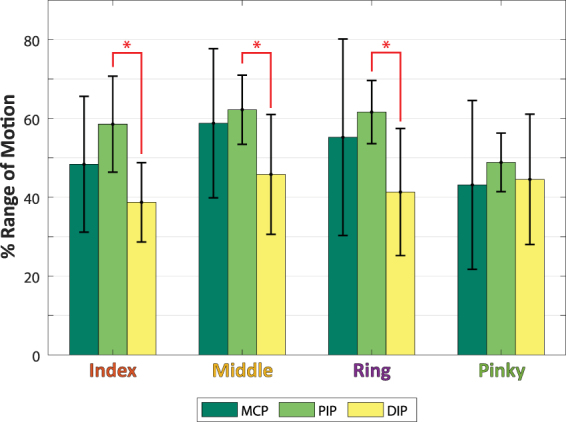
Average Percent of Range of Motion between Fingers and Joints. Error bars represent standard deviation, and asterisks represents statistical significance. All joints within and between fingers showed no significant difference in the range of motion except between the PIP and DIP within each of the Index, Middle, and Ring Fingers.

We also examined whether there was differential movement across different joints of a finger. Figure 6 shows the average range of motion across all trials in Cluster 1. The sphericity assumption of the ANOVA was evaluated with Mauchly’s sphericity test, and given that the assumption of equal variance was violated (*p* < 0.05), a Greenhouse-Geissner correction was performed during the test. The ANOVA showed a significant interaction between the joint and different finger motions [F(2.67, 50.675) = 4.612, *p* = 0.008]. Further post-hoc pairwise comparisons of the individual joints showed that only the PIP and DIP joints of the first three fingers (Index, Middle, and Ring) were significantly different from each other (*p* < 0.05). All other joints showed no significant difference.

## Discussion

This study sought to explore the feasibility of transcutaneous proximal nerve stimulation in the activation and control of different finger motions. Electrical stimulation of the median and ulnar nerves proximal to the elbow resulted in varying hand grasp patterns, which were recorded using a motion tracking system and a simplified joint-finger model. Our results showed that different single finger and multi-finger motions could be elicited using this non-invasive stimulation method. Variations in the stimulation location have shown to be able to change the grasp patterns of the finger joints. Our findings demonstrate the feasibility of a novel stimulation approach that can potentially be used as an assistive or rehabilitative approach, in order to elicit various finger grasp patterns for individuals with weakness in their hand muscles.

Some of the finger movements elicited with nerve stimulation in this study are generally similar to those elicited in previous literature^12,13^. The whole hand power grasp is often reported as a commonly recruited grasp patterns in neuromuscular FES^7^, and our results show this grasp pattern to also be commonly recruited through transcutaneous nerve stimulation. Additionally, our methodology also demonstrated selective multi-finger motions which were stable with a constant stimulation location. These observed finger grasp patterns are likely due to the selective recruitment of different limited numbers of fibers in the median and ulnar nerve bundles. Generally speaking, the median and ulnar nerves are organized so that each innervates roughly half of the finger flexors, divided by the left and right sides. The multi-finger patterns observed from this study seem to follow this distribution of the two nerves, with regions of functional overlap. For example, the clusters of finger motions exist between the Index/Middle fingers (Median), Index/Middle/Ring fingers (Median + Ulnar), and the Middle/Ring/Pinky fingers (Ulnar), but not fingers on the opposite sides of the hand, e.g. Index/Pinky. By changing the stimulation location, differential amounts of charge are delivered to one or both of the nerve bundles based on its relative proximity to each. This makes it possible to control the degree of recruitment of the nerve fibers innervating different finger muscles.

Although the functional quantification of movements solely based on joint angles does not provide a complete picture of movement quality, it is important to note that some of the elicited movement patterns in this study were relevant to activities of daily living. As mentioned, the whole hand grasp was the most common pattern seen across subjects, and is also an important movement of the hand in a majority of tasks involving object manipulation or transport. Aside from this simple pattern, another functionally relevant movement pattern we commonly observed was the finger pinch (Fig. 4 Cluster 4-4, Fig. 5D, Supplementary Video). This movement is used in a variety of dexterous tasks such as key locks or utensil use. Lastly, the single finger motions observed are especially relevant to tasks like typing which is an increasingly important task in daily life today. The methodology proposed in this study requires significant development prior to clinical applications, but it is promising in its potential to target and strengthen specific, functionally relevant movements of the fingers.

Physiologically, neuromuscular stimulation through electrode pads placed on the muscle belly largely activates the superficial muscles close to the skin. In order to access the deeper muscles away from the skin, larger stimulus amplitudes are required, which typically leads to more diffused recruitment of muscles, further limiting the selectivity of muscle activation. On the other hand, given the stimulation only targets extrinsic hand muscles due to the nature of electrode placement, the intrinsic hand muscles in the palmer side of the hand are typically out of reach, although those intrinsic muscles are also actively engaged during natural voluntary grasping^11^. Comparatively, with a relatively low current intensity (<5 mA and 100 µs pulse width), proximal nerve stimulation has the potential to activate nerve fibers that innervate deep muscles as well as intrinsic hand muscles. This potentially can lead to more natural grasp patterns. However, further studies using muscle imaging or electromyogram recordings are necessary to test these possibilities.

The variety of grasp patterns seen in our study were all generated with stimulation trains with current 4.23 ± 1.22 mA at a constant 100 µs pulse duration. These parameters used for our proximal nerve stimulation are substantially lower than traditional values used in previous neuromuscular stimulation approaches that typically range from 10–20 mA with 200–300 µs^13,18^. Such a high current intensity is needed even with careful placement of electrodes over the innervation points of the muscle. This is largely due to the fact that after branching out of the bundles, the distal portion of the nerve fibers can still branch further out and travel into different portions/depth of the muscle. As a result, to activate sufficient muscle fibers that can produce meaningful force output, a high current intensity is inevitable. In contrast, at the nerve bundles proximal to the elbow, the axons innervating all the hand flexor muscles are in close proximity, and the median and ulnar nerves are superficial relative to the skin. Therefore, with a low stimulation current, we can activate a large number of nerve fibers innervating different muscles to a similar extent.

The low current input in our approach can lead to a two-fold benefit in both subject comfort and reduced power consumption for devices. At typically higher currents and longer pulse durations, electrical stimulation is often associated with a sharp noxious sensation, which is uncomfortable or even painful to the recipient, limiting user adoption of electrical stimulation systems. In addition to avoiding stimulation discomfort, a reduced current amplitude and pulse width can reduce the overall power consumption of such a stimulation system. This can also potentially benefit future development of a portable stimulation system designed for the peripheral nerve, as it will be able to operate for a longer time.

With further development, this non-invasive stimulation method could be utilized as a therapeutic tool in aiding individuals with weakened hand muscles to regain strength and function, such as in stroke, spinal cord injury or cerebral palsy populations. Access to both deep and superficial muscles can lead to generating more natural movements with less discomfort and reduced fatigue. These factors would enable this proximal nerve stimulation technique to be a more desirable rehabilitative tool than the more traditional neuromuscular FES methods. Additionally, if combined with user-feedback and control, the stimulation could be designed to reinforce motion intentions and potentially enhance neural plasticity of the central nervous system to further amplify the therapeutic effect^18^. Lower stimulation intensity and slower fatiguing of the muscles due to H-reflex activation^19^ would allow for more prolonged use and greater overall benefit to the users.

As our current study was the first attempt to demonstrate the proof-of-principle of transcutaneous proximal nerve stimulation on finger control, we encountered a few limitations. The first limitation that requires investigation is to test the stability of a single stimulation location in eliciting the same motion pattern over time. Although locating and precisely securing stimulation positions were a time-consuming portion of our experimental setup, anecdotally, stimulation over the same region of skin without any major movement of the electrode resulted in consistent motion patterns. A potential solution for a more secure and efficient method of stimulation would be to utilize a multi-channel stimulation electrode array, which can allow us to activate different fibers in each bundle from different electrode pairs and to activate similar fibers from different electrode pairs, such that this built in redundancy can compensate for the electrode shift. With this multi-electrode setup, a more stringent test of the consistency and stability of a stimulation “location” can be investigated to ensure the repeatability of targeted hand motions. Secondly, our joint marker model greatly simplifies the total motion of the hand while ignoring any abduction/adduction of the fingers as well as the complexity of the carpometacarpal joint of the thumb. In addition, the kinematics of the fingers also do not elucidate how much force can be generated from our stimulation paradigm. Further investigation on the selectivity and control of the force levels in different gripping patterns will also be required to investigate the feasibility of our method. Another limitation of our present study was our exclusion of the wrist joint. During our search of hand electrode positions, we did observe wrist flexion on occasion. However, the electrode position was typically selective to the wrist or the fingers at the current level we tested. Higher current could potentially lead to a more global activation of the finger and wrist flexors concurrently. The evoked wrist flexion could be beneficial to the intended motion, if a wrist flexion is required, or if a strong whole hand grasp and a wrist flexion is needed. However, if a strong hand grasp without wrist motion is needed, the high current stimulation could lead to unwanted dual contractions.

This study demonstrated dexterous control of the finger motions via non-invasive electrical stimulation of the proximal nerves. This non-traditional stimulation method shows promise in being a feasible way to generate selective hand motions for both assistive and rehabilitative purposes while potentially alleviating some of the difficulties faced by other currently available FES methods.

## Electronic supplementary material

Supplemental Video 1

Supplemental Video 2

Supplemental Video 3

Supplemental Video 4

Supplemental figures
