# Supplementary figures and images for: Exploration of Hand Grasp Patterns Elicitable Through Non-Invasive Proximal Nerve Stimulation
Source: Sci Rep. 2017 Nov 29;7:16595. doi: 10.1038/s41598-017-16824-1 (PMC5707381; doi:10.1038/s41598-017-16824-1)

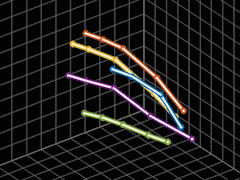

Supplement: Supplementary file 1 — Supplemental Video 1 [file 41598_2017_16824_MOESM1_ESM.gif]

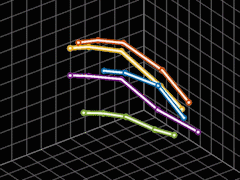

Supplement: Supplementary file 2 — Supplemental Video 2 [file 41598_2017_16824_MOESM2_ESM.gif]

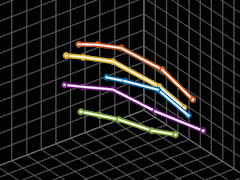

Supplement: Supplementary file 3 — Supplemental Video 3 [file 41598_2017_16824_MOESM3_ESM.gif]

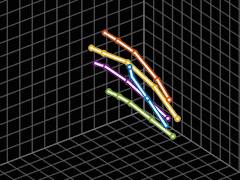

Supplement: Supplementary file 4 — Supplemental Video 4 [file 41598_2017_16824_MOESM4_ESM.gif]
