# Supplementary material for: Exploration of Hand Grasp Patterns Elicitable Through Non-Invasive Proximal Nerve Stimulation
Source: Sci Rep. 2017 Nov 29;7:16595. doi: 10.1038/s41598-017-16824-1 (PMC5707381; doi:10.1038/s41598-017-16824-1)
Supplement: Supplementary file 5 — Supplemental figures [file 41598_2017_16824_MOESM5_ESM.pdf]

Exploration of Hand Grasp Patterns Elicitable Through Non-Invasive Proximal Nerve Stimulation

Henry Shin[1], Zach Watkins[1], and Xiaogang Hu[1]

[1]Joint Department of Biomedical Engineering, University of North Carolina at Chapel Hill, NC and North Carolina State University, Raleigh, NC

Supplementary Figures

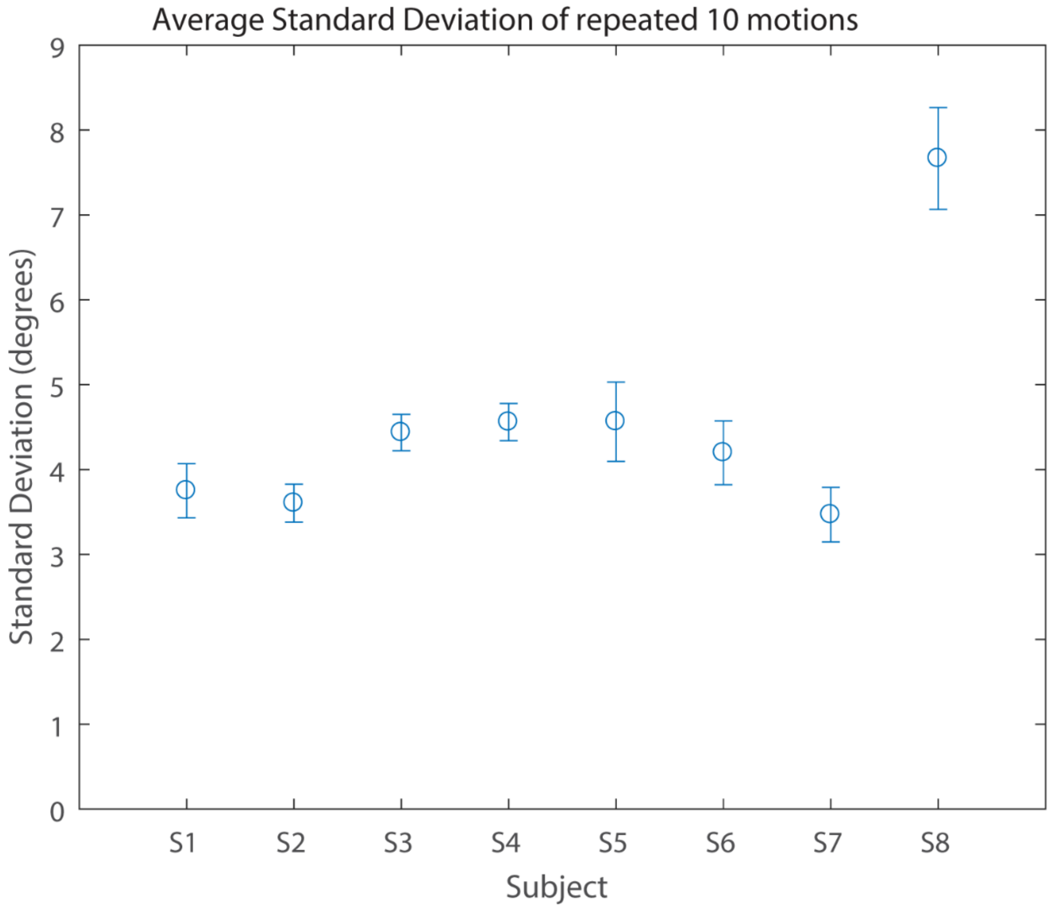

Figure S1: Average Standard Deviation of each trial for each subject. The standard deviation of the 10 repeated motions of each trial was calculated and binned based on the subject to get a pooled mean (circle) and standard error (bars) across all trials for each subject

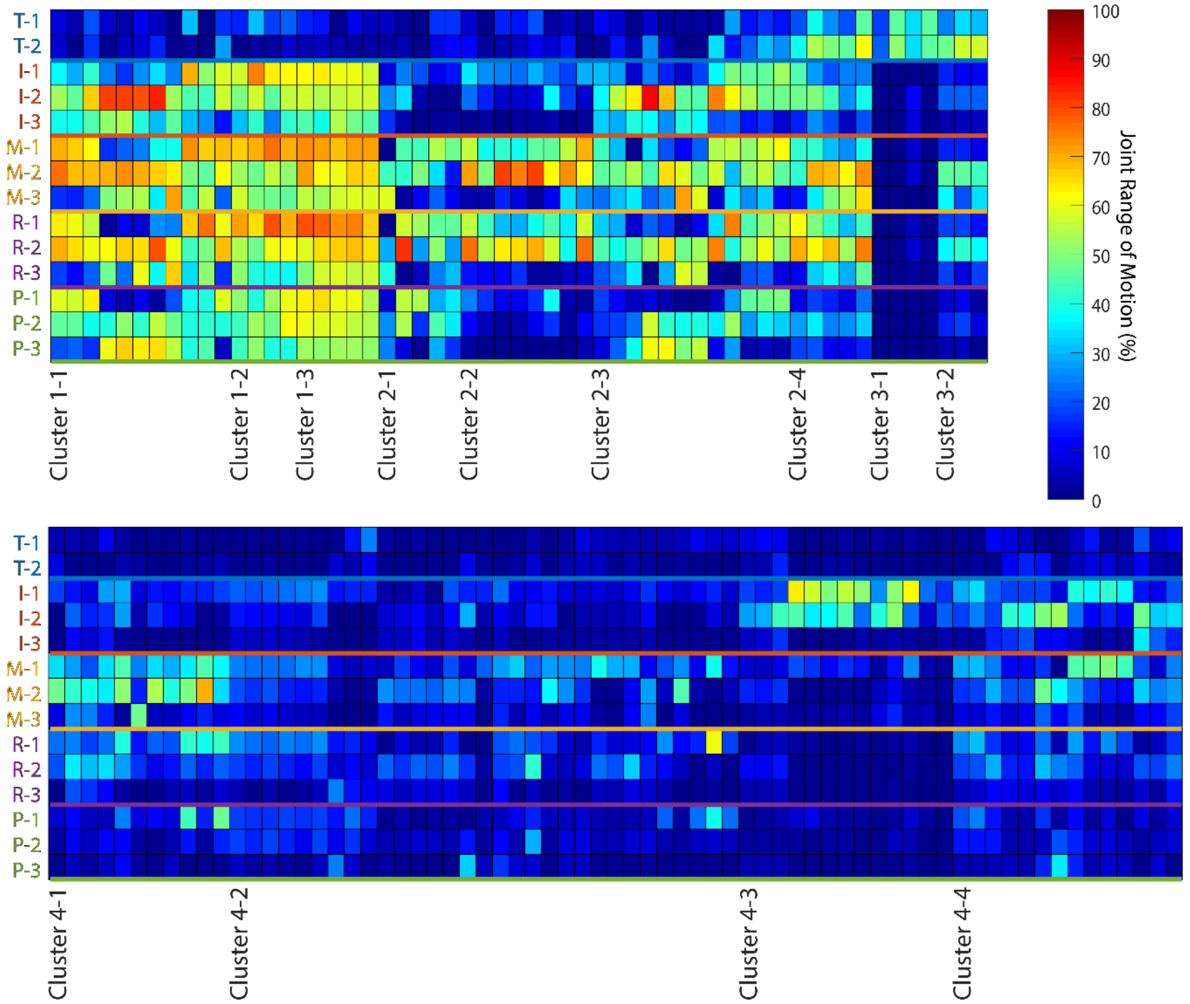

Figure S2: Clustered Finger Joint Motions. The two levels of k-means clustering from Figure 4 in the main text was expanded to see all of the individual joints which were averaged to estimate the average motion of the whole finger. Cluster numbers (Main # - Sub #) indicate the first column (trial) that was categorized into the cluster.
